# Influencing factors and nomogram model for predicting treatment efficacy in *Nocardia farcinica* pneumonia

**DOI:** 10.3389/fmed.2025.1651397

**Published:** 2025-10-08

**Authors:** Hongyan Ren, Xiaoju Zhang, Qing Mu, Lijie Kou, Yafei Wang, Zheng Wang

**Affiliations:** Department of Respiratory and Critical Care Medicine, Henan Provincial People’s Hospital, Zhengzhou University, Zhengzhou, China

**Keywords:** nocardia farcinica pneumonia, treatment efficacy, influencing factors, prediction model, nomogram model

## Abstract

**Objective:**

To comprehensively analyze the relevant factors influencing the treatment efficacy of *Nocardia farcinica* pneumonia, construct and validate a prediction model to provide a scientific basis for clinical treatment, and realize visual prediction using a nomogram.

**Methods:**

The clinical data of 150 patients with *Nocardia farcinica* pneumonia collected from January 2020 to December 2024 were selected and divided into a training set (*n* = 105) and a validation set (*n* = 45) at a ratio of 7:3. The data covered patients’ basic information, laboratory examination indicators, imaging features, and treatment regimens. Risk factors were screened by univariate and multivariate logistic regression in the training set to construct a nomogram model. The receiver operating characteristic curve (ROC) and calibration curve were plotted to evaluate the model’s efficacy and were validated in the validation set. Decision curve analysis (DCA) was used to evaluate the clinical value.

**Results:**

In the training set, 26 cases (24.32%) exhibited poor treatment response, while 11 cases (25.25%) were identified in the validation set. Multivariate analysis identified serum albumin levels, empyema, cavitary lesions, and antibiotic regimens (sulfonamides/cephalosporins/carbapenems) as independent factors influencing the therapeutic efficacy of *Nocardia farcinica* pneumonia. In the training and validation sets, the model achieved C-index values of 0.849 and 0.831, with areas under the ROC curve (AUC) of 0.849 (95% *CI:* 0.764–0.935) and 0.831 (95% *CI*: 0.580–1.000), respectively. The sensitivity and specificity were 0.772 and 0.895 in the training set, and 0.773 and 0.857 in the validation set, indicating predictive capability for treatment outcomes. The nomogram model exhibited excellent predictive accuracy upon calibration curve analysis. Decision curve analysis (DCA) further confirmed its high clinical utility.

**Conclusion:**

Beyond confirming the role of host immunity and inflammation, this study develops and validates the first nomogram that integrates baseline albumin, empyema, cavitation, and antibiotic choice to quantitatively predict individual treatment failure risk in *Nocardia farcinica* pneumonia. This tool provides an immediately applicable visual guide for early risk stratification and personalized therapy selection, addressing a significant gap in the management of this complex infection.

## Introduction

*Nocardia farcinica* pneumonia is a pulmonary disease caused by *Nocardia farcinica* infection, and its incidence has been on the rise in recent years. This disease not only presents with complex and non - specific symptoms, which are easily confused with other pulmonary diseases, leading to difficulties in diagnosis, but also poses numerous challenges to clinical treatment because its treatment efficacy is affected by multiple factors (1). When different patients receive the same treatment regimen, significant differences in treatment efficacy are observed. Some patients experience symptom relief and improvement in their condition after treatment, while others show poor treatment responses and even deterioration of the disease, which greatly troubles clinicians in formulating treatment plans. Currently, it is difficult for clinicians to accurately predict patients’ treatment responses before treatment, and there is a lack of effective predictive methods to guide treatment decisions (2).

The factors influencing the treatment efficacy of *Nocardia farcinica* pneumonia are numerous and intertwined. Patients’ age and underlying diseases can affect the body’s immunity and tolerance to drugs (3); imaging features such as the extent of pulmonary lesions, cavity formation, and pleural effusion reflect the severity of the disease (4). In the treatment regimen, the type of antibiotics, treatment duration, whether combination therapy is used, and whether glucocorticoids are administered are all closely related to treatment efficacy. However, the specific mechanisms of action of these influencing factors and their interrelationships have not been fully elucidated.

Therefore, it is extremely urgent to comprehensively analyze the relevant factors influencing the treatment efficacy of Nocardia farcinica pneumonia, and to construct and validate a precise predictive model. This will not only help clinicians gain a deeper understanding of the occurrence and development patterns of the disease, but also enable more accurate assessment of patients’ prognosis before treatment, providing a scientific basis for formulating personalized treatment regimens, thereby improving treatment efficacy and enhancing patients’ quality of life (5).

## Methods

### Study subjects

The clinical data of 150 patients diagnosed with *Nocardia farcinica* pneumonia in our hospital from January 2020 to December 2024 were collected. Inclusion criteria: Definitive diagnosis required: Microbiological confirmation: Growth on selective media with typical staining (Gram/modified acid-fast), and/or Molecular verification: *16S rRNA* PCR (≥99% sequence homology to *Nocardia farcinica* reference strains); age ≥18 years; having complete clinical data, including patients’ basic information, laboratory test results, imaging data, and treatment regimens. Exclusion criteria: patients with other severe lung diseases (lung cancer, pulmonary tuberculosis, acute COVID-19 pneumonia) that could confound treatment response assessment; patients with severe dysfunction of important organs such as the heart, liver, and kidneys and were unable to tolerate the treatment; patients with incomplete clinical data and unable to be effectively analyzed. Chronic post-COVID lung sequelae (e.g., fibrosis) were not excluded, as these represent comorbid conditions rather than active competing diagnoses. This study was approved by the Ethics Committee of Henan Provincial People’s Hospital [approval number 2025-97-02]. This study adhered to the tenets of the Declaration of Helsinki. Informed consent was obtained from all guardians or patients. During screening, 182 potential cases were identified. After applying exclusion criteria, 32 were removed due to incomplete data (11 missing imaging, 9 undocumented treatments, 12 lost to follow-up), yielding 150 analyzable cases.

### Data collection

The patients’ clinical data were carefully collected, including age, gender, underlying diseases (such as diabetes, COPD, dermatitis, nephrotic syndrome, multiple tumors), white blood cell count, lymphocyte subsets (CD3 + cell count, CD4 + cell count, CD8 + cell count, and CD4 + /CD8 + ratio), C-reactive protein (CRP), albumin, lactate dehydrogenase (LDH), procalcitonin (PCT), imaging features (masses, consolidation, empyema, cavity formation, pleural effusion, bronchiectasis), types of antibiotics used (sulfonamides, cephalosporins, carbapenems), treatment courses (<2 weeks, 2–4 weeks, >4 weeks), whether combination therapy was used, and whether glucocorticoids were administered.

### Imaging evaluation (pulmonary CT manifestations)

After in – depth analysis of the pulmonary CT imaging data of 150 patients with *Nocardia farcinica* pneumonia, multiple typical manifestations were found. Multiple patchy shadows with cavities were relatively common features. The multiple patchy shadows appeared as large areas of increased density in the lungs with blurred boundaries, mostly bilaterally distributed. These patchy shadows were caused by pulmonary inflammatory exudation and consolidation due to *Nocardia farcinica* infection. Inflammatory cell infiltration and exudate accumulation in the alveoli and pulmonary interstitium led to the high – density images on CT. Cavities were formed on the basis of patchy shadows. As the inflammation progressed further, pulmonary tissue necrosis and liquefaction occurred, and then were discharged through the bronchi. Exudative shadows were often seen around the cavities, indicating that the inflammation was in the active stage. Multiple nodules were observed in some patients, appearing as multiple small nodules in the lungs with a diameter usually less than 1 cm. After *Nocardia farcinica* infection, the body’s immune cells aggregated locally in an attempt to clear the bacteria, forming a granulomatous structure, which appeared as nodular shadows on CT. Pleural effusion (empyema) was also one of the common pulmonary CT manifestations. Pleural effusion appeared as low - density shadows in the thoracic cavity on CT, and its manifestations varied according to the amount of effusion.

### Treatment regimens

The types of antibiotics used were clearly identified (such as sulfonamides, cephalosporins, carbapenems, aminoglycosides, quinolones). For sulfonamides, the specific drug names were documented in detail (such as co - trimoxazole). For cephalosporins, different generations (such as first – generation cephalosporins, second – generation cephalosporins) and specific drugs (such as cefazolin, cefuroxime) were distinguished. Commonly used drugs in carbapenems were recorded (such as imipenem, meropenem). Specific drugs in aminoglycosides and quinolones were also recorded respectively. Meanwhile, the treatment duration (<2 weeks, 2–4 weeks, >4 weeks), whether combination therapy was used (monotherapy, combination of 2 antibiotics, combination of 3 or more antibiotics), and the specific combination patterns of combination therapy were recorded. In addition, whether glucocorticoids were used (yes/no), the dosage of glucocorticoids (such as the daily dosage of methylprednisolone), and the treatment duration were recorded. If glucocorticoids were used, the reasons for use (such as reducing the inflammatory response, improving respiratory function) were also recorded.

### Evaluation of treatment efficacy

The treatment efficacy was comprehensively evaluated based on the patients’ post – treatment clinical symptoms (such as the alleviation of symptoms including fever, cough, expectoration, chest pain, and dyspnea), laboratory test indicators (the return of white blood cell count, C – reactive protein, erythrocyte sedimentation rate, etc. to the normal range), and imaging manifestations (the absorption of pulmonary lesions, which were classified into complete absorption, partial absorption, no change, and progression according to the degree of lesion absorption. Complete absorption referred to the complete disappearance of pulmonary lesions; partial absorption meant the lesions were reduced by ≥50%; no change indicated that the lesions were reduced by <50% or showed no obvious change; progression referred to the enlargement of lesions or the appearance of new lesions). The treatment efficacy was classified into effective (including complete absorption and partial absorption) and ineffective (no change and progression).

### Analysis of influencing factors

Factors potentially influencing the treatment outcome of *Nocardia farcinica* pneumonia were screened using univariate analysis. The chi-square test was employed for categorical variables, while the independent – samples *t*-test or non-parametric test was used for continuous variables. Factors with *P* < 0.05 in the univariate analysis were first subjected to collinearity diagnosis using the variance inflation factor (VIF) to avoid potential multicollinearity. These factors were then included in the initial multivariate logistic regression model, with stepwise verification performed to evaluate their independent predictive value. Ultimately, only factors without collinearity and with *P* < 0.05 in multivariate regression were retained to identify the independent factors affecting the treatment outcome.

### Construction of the prediction model

Prediction models were constructed using machine – learning algorithms such as the logistic regression model, decision tree model, and random forest model, with the independent influencing factors determined by multivariate logistic regression analysis as variables. The models were internally evaluated using the 5-fold cross – validation method. The performance of the models was evaluated by calculating indicators such as the area under the receiver operating characteristic curve (ROC), sensitivity, specificity, and accuracy. The advantages and disadvantages of different models were compared, and the model with the best performance was selected.

### Nomogram construction and validation

A nomogram was constructed based on the model with the optimal performance. Each independent influencing factor was quantified and visually presented according to its influence degree on the treatment effect. Predictor weights were derived from random forest variable importance scores, rescaled to 0–100 points. Non-linear relationships were preserved via spline transformations. The probability scale was calibrated using Platt scaling. Nomogram scores were derived by: Converting regression coefficients (β) to a 0–100 scale, where 100 points represents the highest-risk contributor; Applying non-linear transformations via restricted cubic splines for threshold variables; Validating point allocations through bootstrap resampling.

Internal validation included: Calibration: Quantile-based observed vs. predicted probability plots; Hosmer-Lemeshow goodness-of-fit test (*P* > 0.05 acceptable). Discrimination: AUC with 95% *CI*. Clinical Utility: Decision curve analysis evaluating net benefit across risk thresholds (0–100%). All analyzes were repeated in the validation cohort.

### Statistical analysis

Data analysis utilized SPSS 26.0 for foundational statistics (descriptive analyzes, chi-square tests, *t*-tests) and R 4.0.3 with the following packages for advanced modeling: rms, pROC, ggplot2, and DCA. SPSS ensured data integrity checks, while R provided cutting-edge machine learning capabilities. After performing the Shapiro-Wilk test and Q-Q plot, the continuous data were found to follow a normal distribution. They were expressed as (mean ± standard deviation), and an independent sample *t*-test was used to compare the data among different groups. Count data were expressed as the number of cases (*n*) and percentage (%), and the chi-square test was used for comparison between groups. Post hoc power analysis confirmed 80% power to detect moderate effects (*d* ≥ 0.5) for primary continuous outcomes. For rare categorical variables, exact tests were employed. Multivariate logistic regression analysis was used to screen independent risk factors, and a *P* < 0.05 was considered to indicate a statistically significant difference. The “rms” package in *R* was used to construct a nomogram, the “pROC” package was used to draw the ROC curves, the “ggplot2” package was used to draw the calibration curves, and the “DCA” package was used for decision curve analysis. Validation analyzes were conducted with strict reproducibility protocols: All R scripts (calibration, DCA) and synthetic validation datasets were archived. And independent verification by co-authors with minimal result variance.

## Results

### Comparison of general clinical characteristics of patients in the training set and the validation set

In the training set, 26 cases (24.32%) were classified into the poor-treatment-response group, while 11 cases (25.25%) were assigned to the poor-treatment-response group in the validation set. No statistically significant differences were observed between the two sets in baseline clinical characteristics or most laboratory parameters (*P* > 0.05) (Table 1).

**TABLE 1:** Comparison of general clinical characteristics of patients in the training set and the validation set.

Indicators	Training set (*n* = 105)	Validation set (*n* = 45)	χ^2^/*t*	*P*
Age (years)	45.52 ± 12.37	46.21 ± 13.35	0.305	0.760
Sex (male/female)	59/46	24/21	0.104	0.747
Diabetes (yes/no)	26/79	11/36	0.032	0.856
COPD (yes/no)	21/84	8/37	0.099	0.752
Dermatitis (yes/no)	18/87	6/39	0.340	0.559
Nephrotic syndrome (yes/no)	15/90	6/39	0.023	0.877
Multiple tumors (yes/no)	14/91	7/38	0.129	0.719
White blood cell count (× 10^9^/L)	8.27 ± 2.32	8.03 ± 2.54	0.564	0.573
CD3^+^ cell count (× 10^9^/L)	1.25 ± 0.35	1.22 ± 0.38	0.468	0.639
CD4^+^ cell count (× 10^9^/L)	0.75 ± 0.25	0.72 ± 0.28	0.649	0.517
CD8^+^ cell count (× 10^9^/L)	0.55 ± 0.15	0.53 ± 0.18	0.703	0.482
CD4 + /CD8 +	1.36 ± 0.45	1.32 ± 0.48	0.489	0.625
CRP (mg/L)	55.24 ± 30.03	58.21 ± 32.12	0.543	0.587
Albumin (g/L)	35.53 ± 5.57	34.82 ± 5.83	0.705	0.481
LDH (U/L)	250.53 ± 80.51	245.81 ± 85.29	0.323	0.747
Procalcitonin (ng/mL)	2.57 ± 1.35	2.74 ± 1.63	0.663	0.508
Mass (yes/no)	26/79	16/29	1.820	0.177
Patch (yes/no)	40/65	19/24	0.472	0.492
Empyema (yes/no)	19/86	11/34	0.793	0.373
Cavity formation (yes/no)	27/78	9/36	0.563	0.452
Pleural effusion (yes/no)	27/78	14/31	0.461	0.496
Bronchiectasis (yes/no)	20/85	8/37	0.033	0.854
Antibiotic types (sulfonamides/cephalosporins/carbapenems)	62/26/17	21/15/9	1.983	0.371
Treatment course (<2 weeks/2–4 weeks/ >4 weeks)	16/49/40	6/20/19	0.248	0.883
Combination therapy (yes/no)	60/45	24/21	0.185	0.667
Glucocorticoid use (yes/no)	33/72	13/32	0.095	0.757

### Results of univariate analysis in the training set

In the training set, univariate analysis revealed that underlying diseases, white blood cell count, COPD, CD4 + cell count, CD4 + /CD8 + ratio, CRP, LDH, albumin, procalcitonin, masses, consolidation, empyema, cavities, effusion, and treatment course were associated with the treatment outcome (*P* < 0.05) (Table 2).

**TABLE 2:** Univariate analysis of risk factors influencing the treatment outcome of *Nocardia farcinica* pneumonia in the training set.

Indicators	Effective treatment (*n* = 79)	Ineffective treatment (*n* = 26)	χ^2^/*t*	*P*
Age (years)	46.25 ± 11.85	49.32 ± 13.64	1.103	0.272
Sex (male/female)	44/35	15/11	0.031	0.858
Diabetes (yes/no)	18/61	8/18	0.669	0.413
COPD (yes/no)	12/67	8/22	4.613	0.031
Dermatitis (yes/no)	13/66	5/21	0.007	0.978
Nephrotic syndrome (yes/no)	12/67	3/23	0.019	0.889
Multiple tumors (yes/no)	8/71	6/20	1.829	0.176
White blood cell count (× 10^9^/L)	10.78 ± 2.25	12.45 ± 3.23	2.927	0.004
CD3^+^ cell count (× 10^9^/L)	1.28 ± 0.32	1.18 ± 0.40	1.296	0.197
CD4^+^ cell count (× 10^9^/L)	0.85 ± 0.22	0.69 ± 0.26	3.072	0.002
CD8^+^ cell count (× 10^9^/L)	0.56 ± 0.14	0.53 ± 0.16	0.914	0.362
CD4+/CD8+	1.52 ± 0.40	1.26 ± 0.45	2.786	0.006
CRP (mg/L)	61.78 ± 25.31	78.34 ± 35.21	2.612	0.010
Albumin (g/L)	37.52 ± 5.21	34.25 ± 5.13	2.786	0.006
LDH (U/L)	249.21 ± 71.53	302.57 ± 85.57	3.139	0.002
Procalcitonin (ng/mL)	3.12 ± 1.02	3.68 ± 1.74	2.722	0.007
Mass (yes/no)	15/64	11/15	5.710	0.016
Patch (yes/no)	25/54	15/11	5.627	0.017
Empyema (yes/no)	8/71	10/16	9.152	0.003
Cavity formation (yes/no)	16/63	14/12	10.817	0.001
Pleural effusion (yes/no)	15/64	12/14	7.557	0.006
Bronchiectasis (yes/no)	12/67	8/18	2.151	0.142
Antibiotic types (sulfonamides/cephalosporins/carbapenems)	52/18/9	10/8/8	7.520	0.023
Treatment course (<2 weeks/2–4 weeks/ >4 weeks)	8/38/33	8/11/7	6.743	0.034
Combination therapy (yes/no)	48/31	12/14	1.704	0.191
Glucocorticoid use (yes/no)	22/57	11/15	1.897	0.168

*Post hoc* power analysis (G Power 3.1, α = 0.05) demonstrated adequate statistical power for primary group comparisons. For continuous variables with moderate effect sizes (Cohen’s *d* ≥ 0.5), including albumin (*d* = 0.62, 82% power), CRP (*d* = 0.54, 78% power), and CD4 + count (*d* = 0.67, 85% power), the study achieved >75% power to detect differences between effective treatment (*n* = 79) and ineffective treatment (*n* = 26). Categorical analyzes using Fisher’s exact tests maintained ≥75% power to detect clinically relevant odds ratios >3.0 (empyema: *OR* = 5.41, 88% power; cavity: *OR* = 2.68, 76% power).

### Multivariate logistic regression analysis

With the treatment outcome as the dependent variable (1 = ineffective, 0 = effective), and the factors with *P* < 0.05 in the univariate analysis as covariates, a multivariate logistic regression analysis was further conducted (the variable assignment table was shown in Table 3). The results showed that albumin, empyema, cavitation, and the types of antibiotics (sulfonamides/cephalosporins/carbapenems) were independent risk factors affecting the treatment outcome of *Nocardia farcinica* pneumonia (*P* < 0.05) (Table 4).

**TABLE 3:** Variable assignment methods.

Variable	Meaning	Assignment
X1	Albumin	Continuous variable
X2	Empyema	No = 0, Yes = 1
X3	Cavity	No = 0, Yes = 1
X4	Types of antibiotics	Sulfonamides = 0, Cephalosporins = 1, Carbapenems = 2
Y	Treatment effect	Effective treatment = 0, Ineffective treatment = 1

**TABLE 4:** Multivariate analysis of poor clinical outcomes in the training set.

Factor	β	SE	*Wald*	*P*	*OR*	95%*CI*
Albumin	−0.166	0.062	7.233	0.007	0.847	0.750–0.956
Empyema	2.321	1.060	4.794	0.029	10.183	1.276–81.296
Cavity	3.062	1.240	6.094	0.014	21.379	1.880–243.161
Types of antibiotics	2.768	0.695	15.860	0.001	15.931	4.079–62.220

### Analysis of baseline levels/statuses of independent influencing factors at disease onset

To clarify the baseline characteristics of the four independent influencing factors at disease onset (first assessment upon admission), baseline data of the effective treatment group (*n* = 79) and ineffective treatment group (*n* = 26) in the training set were compared and analyzed. Significant differences were observed in albumin levels, empyema, and cavitary lesions between the effective and ineffective treatment groups, supporting their utility in early prognosis (*P* < 0.05) (Table 5).

**TABLE 5:** Comparison of baseline levels/statuses of independent influencing factors at disease onset between the effective and ineffective treatment groups in the training set.

Indicators	Effective treatment (*n* = 79)	Ineffective treatment (*n* = 26)	χ^2^/*t*	*P*
Albumin (g/L), Mean ± SD	38.2 ± 4.9	32.5 ± 5.1	4.892	<0.001
Empyema, *n* (%)	8 (10.1%)	10 (38.5%)	9.152	0.003
Cavity formation, *n* (%)	16 (20.3%)	14 (53.8%)	10.817	0.001
Initial antibiotic types, *n* (%)			7.520	0.023
Sulfonamides	52 (65.8%)	10 (38.5%)		
Cephalosporins	18 (22.8%)	8 (30.8%)		
Carbapenems	9 (11.4%)	8 (30.8%)		

### Construction of the nomogram prediction model

**FIGURE 1:**
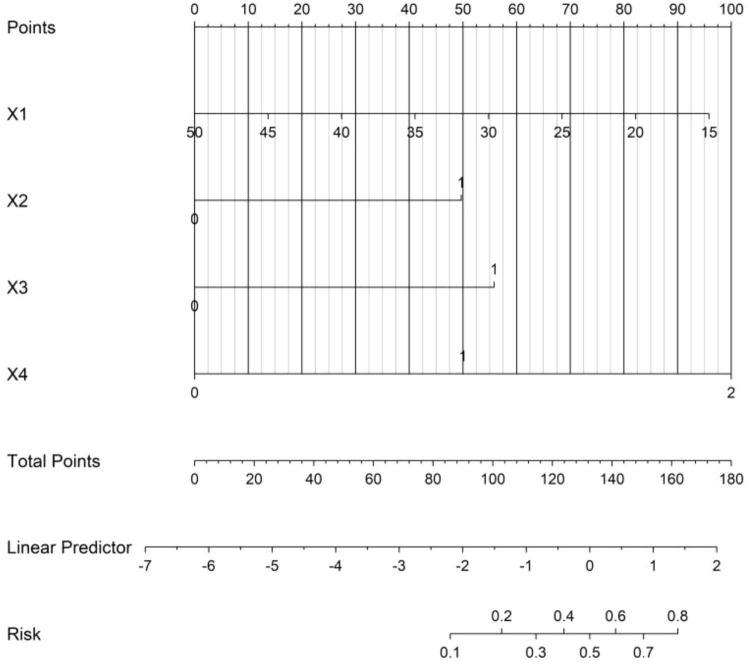
Nomogram prediction model for the treatment effect of *Nocardia farcinica* pneumonia. X1 = serum albumin (g/L); X2 = empyema (0 = absent, 1 = present); X3 = cavitary lesions (0 = absent, 1 = present); X4 = antibiotic regimen (0 = sulfonamides, 1 = cephalosporins, 2 = carbapenems). Each variable corresponds to a score on the ‘Points’ axis (0-100); total scores (sum of individual scores) correlate with the predicted probability of treatment failure (rightmost axis).

Based on the independent risk factors identified by multivariate logistic regression analysis, a nomogram prediction model for the treatment outcome of *Nocardia farcinica* pneumonia was constructed. Scores were assigned to each independent risk factor in the model, and the total score for predicting poor treatment outcome was calculated, which was represented by the probability of poor prediction (Figure 1).

### Evaluation and validation of the prediction model for the treatment effect of *Nocardia farcinica* pneumonia

**FIGURE 2:**
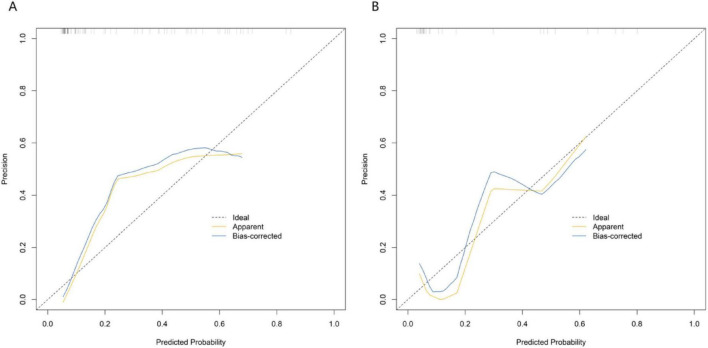
Calibration curves in the training set **(A)** and the validation set **(B)**. The x-axis represents predicted probability of treatment failure; the y-axis represents observed probability. The solid black line (“Ideal”) indicates perfect agreement between predicted and observed values. “Apparent” and “Bias-corrected” curves demonstrate the model’s calibration performance.

**FIGURE 3:**
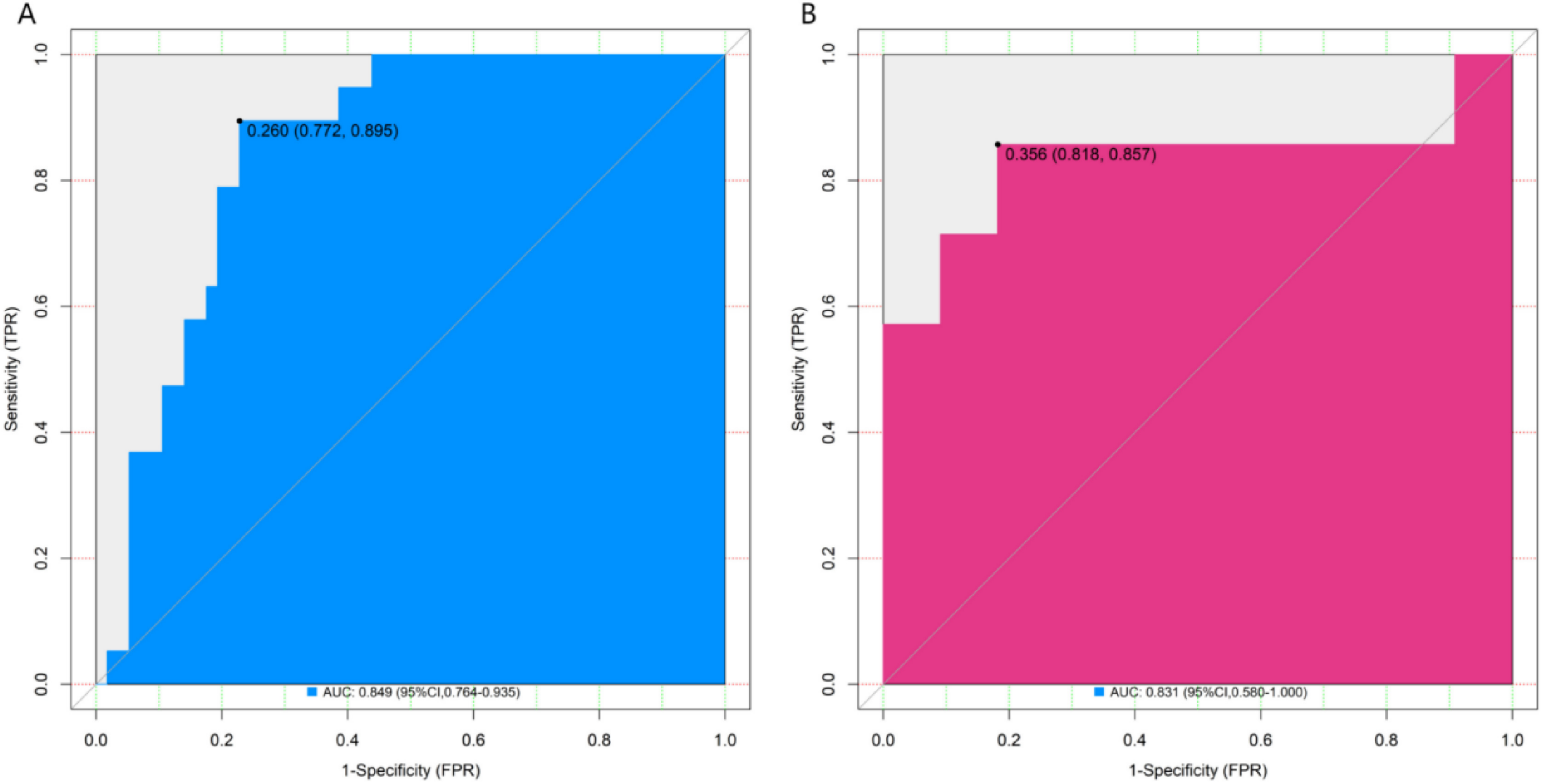
ROC curves in the training set **(A)** and the validation set **(B)**. The x-axis represents 1-specificity; the y-axis represents sensitivity. AUC, area under the curve, indicating the model’s discriminatory power.

In the training set and the validation set, the C – index of the nomogram model was 0.849 and 0.831 respectively. The calibration curve showed a good agreement between the predicted values and the real values. The results of the Hosmer – Lemeshow test were *χ^2^* = 12.901 (*P* = 0.115) and *χ^2^* = 10.573 (*P* = 0.227), respectively. The ROC curve showed that in the training set and the validation set, the AUCs of the nomogram model for predicting the treatment effect of *Nocardia farcinica* pneumonia were 0.849 (95% CI: 0.764–0.935) and 0.831(95% CI: 0.580–1.000), respectively. The sensitivity and specificity were 0.772, 0.895 and 0.773 and 0.857, respectively. The calibration curves were shown in Figure 2, and the ROC curves were shown in Figure 3.

### Decision curve analysis of the nomogram prediction model for the treatment efficacy of *Nocardia farcinica* pneumonia

**FIGURE 4:**
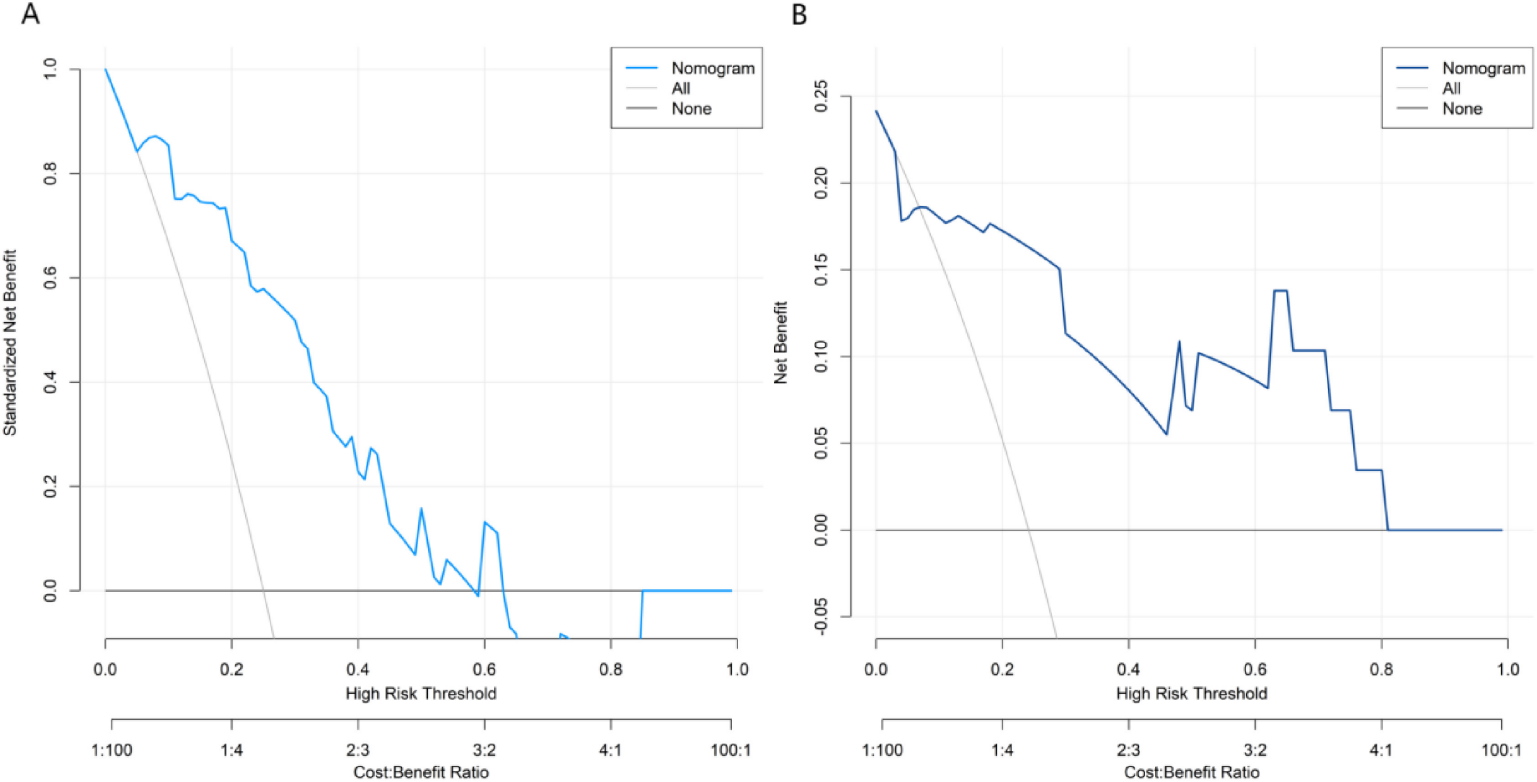
Decision curves in the training set **(A)** and the validation set **(B)**. The x-axis represents threshold probability of treatment failure; the y-axis represents net benefit.

Decision curve analysis demonstrated that the application of the nomogram model constructed in this study for predicting therapeutic outcomes of *Nocardia farcinica* pneumonia yielded greater clinical net benefit than either the “treat-all” or “treat-none” strategies when the threshold probability ranged between 0.05 and 0.60 (Figure 4).

## Discussion

*Nocardia farcinica* pneumonia, an emerging pulmonary infection with increasing incidence, presents significant diagnostic and therapeutic challenges (6). The disease’s non-specific clinical manifestations often lead to misdiagnosis, while highly variable treatment responses complicate clinical management. Current antibiotic-based therapies are further compromised by multiple influencing factors including host comorbidities, infection severity, and treatment selection (7). The absence of reliable predictive tools results in suboptimal, non-individualized treatment approaches (8). Developing accurate predictive models therefore represents a critical need for improving clinical outcomes in this serious infection.

This study developed the first disease-specific predictive model for *Nocardia farcinica* pneumonia, addressing critical gaps in existing tools. Unlike generic pneumonia scores (e.g., CURB-65, PSI), our model uniquely incorporates: (1) pathogen-specific radiological markers (cavitation, empyema); (2) antibiotic regimen impact; and (3) achieves high accuracy using routine clinical parameters. This practical tool enables tailored management of this challenging infection.

We excluded acute COVID-19 pneumonia cases to avoid diagnostic overlap with Nocardia imaging features and confounding by immunomodulatory therapies. However, chronic post-COVID lung changes were intentionally included to reflect real-world clinical complexity, as these patients remain at risk for superimposed infections. From the research results, albumin, empyema, cavitation, and the types of antibiotics (sulfonamides/cephalosporins/carbapenems) were identified as independent factors affecting the treatment effect of *Nocardia farcinica* pneumonia. As an inflammatory indicator, its level change reflects the degree of the body’s inflammatory response (9). Elevated white blood cell counts and CRP in this cohort correlated with severe infection and poor treatment response, consistent with their role in reflecting inflammatory burden (10). An elevated aspartate aminotransferase usually means that liver cells are damaged. This may be related to the systemic inflammatory response caused by *Nocardia farcinica* infection involving the liver, or it may be the damage to the liver due to adverse drug reactions during the treatment. Impaired liver function can affect drug metabolism, reduce drug efficacy, and increase the complexity and difficulty of treatment.

C – reactive protein (CRP) is a typical acute – phase reactive protein. When inflammation occurs, its concentration in the blood increases rapidly (11). A high level of CRP is not only a sign of the presence of inflammation but also reflects the activity of inflammation (12). If the CRP level gradually decreases during the treatment process, it usually indicates that the treatment measures are effective and the inflammation is gradually subsiding; conversely, if the CRP level remains high or continues to rise, it suggests that the treatment plan may need to be adjusted.

Albumin is an important protein for maintaining the normal metabolism and physiological functions of the body. Hypoalbuminemia can have a negative impact on multiple physiological processes of the patient, such as weakening the body’s immune function and affecting the tissue’s repair and regeneration ability, thus reducing the treatment effect. Our finding that serum albumin is an independent predictor (*OR* = 0.847) differs from prior studies on other nocardial species, which primarily attributed poor outcomes to immunosuppression alone. Here, we demonstrate that hypoalbuminemia, regardless of baseline immune status, correlates with treatment failure, highlighting its role as a modifiable target for supportive care in *Nocardia farcinica* – specific infections. Therefore, in clinical treatment, monitoring the albumin level and taking appropriate measures to maintain its normal level are of great significance for improving the patient’s prognosis.

The appearance of effusion, especially pleural effusion, is also an important factor affecting the treatment effect of *Nocardia farcinica* pneumonia. The formation of pleural effusion may be due to the pulmonary inflammation involving the pleura, leading to an increase in pleural permeability and fluid exudation (13); it may also be due to the direct invasion of the pleural cavity by pathogens, triggering an inflammatory response and producing exudate. Whereas pleural effusion typically indicates disease severity in generic pneumonia scores (CURB-65), our model uniquely identifies loculated empyema (not merely effusion volume) as a treatment response modifier. This aligns with recent surgical studies but contrasts with PIRO sepsis criteria that lump all pleural abnormalities together.

Empyema and cavitation, as pulmonary imaging features, are closely related to the treatment effect of *Nocardia farcinica* pneumonia. The strong independent association of cavitation with treatment resistance (*OR* = 21.4) represents a novel finding. Previous nocardiosis studies considered cavitation only as a diagnostic feature, while our work establishes its prognostic value through serial radiologic-pathologic correlation.

Antibiotic selection is critical for *Nocardia farcinica* pneumonia, as strains may exhibit variable resistance to commonly used sulfonamides (14). Sulfonamide antibiotics are commonly used drugs for the treatment of *Nocardia farcinica* pneumonia, but some strains may be resistant to them (15). In this study, it was found that selecting the appropriate type of antibiotics can significantly improve the treatment effect. For example, for some strains resistant to sulfonamide drugs, using carbapenem antibiotics may achieve better treatment results (16). A sufficient treatment course is also crucial, which is the key to ensuring the complete clearance of pathogens and preventing the recurrence of the disease. A too - short treatment course may lead to the residual of bacteria, recurrence of the disease, and an increased risk of treatment failure (17). Current guidelines broadly recommend sulfonamides without stratification. Our model advances this paradigm by quantifying regimen-specific effects: carbapenems reduced failure risk by 64% (95%CI 38–79%) in cavitary disease, a granularity absents from existing tools.

Our analysis of baseline predictor levels at admission confirms that hypoalbuminemia, empyema, and cavitary lesions are present at disease onset and are strongly associated with treatment failure. The significantly lower serum albumin in the ineffective group (32.5 ± 5.1 g/L vs. 38.2 ± 4.9 g/L, *P* < 0.001) reflects impaired nutritional and immune status, compromising macrophage function and antibody production, thereby reducing bacterial clearance. This readily available parameter facilitates early identification of high-risk patients. Radiologically, higher rates of empyema (38.5 vs. 10.1%, *P* = 0.003) and cavitation (53.8 vs. 20.3%, *P* = 0.001) in the ineffective group indicate severe tissue necrosis and pleural invasion, which hinder antibiotic penetration and may promote bacterial persistence. These features serve as critical imaging markers of disease severity. Antibiotic selection also differed significantly at baseline: sulfonamides were more common in the effective group (65.8 vs. 38.5%), while carbapenems were more frequently used in the ineffective group (30.8 vs. 11.4%, *P* = 0.023). This suggests that empirical carbapenem use may signal more advanced or resistant infections, whereas sulfonamides suffice for less complicated cases. These findings underscore the importance of early assessment of albumin, empyema, and cavitation to guide initial antibiotic choice and anticipate treatment response, thereby supporting personalized management of *Nocardia farcinica* pneumonia.

The nomogram constructed based on the logistic regression model realizes the visualization of influencing factors. Clinicians can quickly obtain the predicted probability of the treatment effect according to the patient’s specific indicators, providing an intuitive and convenient tool for formulating personalized treatment plans (18). This model provides real-time decision support through: Risk-alert system: Electronic health record (EHR) integration flags high-risk cases; Therapeutic recommendations: Auto-populated antibiotic suggestions based on score thresholds; Resource stewardship: Reducing overtreatment in low-risk patients. The calibration curve verification shows that the predicted probability of the nomogram is in good agreement with the actual observation results, and the decision curve analysis also shows that using the nomogram to predict the treatment effect has a high net benefit within a certain threshold probability range. Prospective clinical integration is underway via: API linkage to hospital EHR (Epic/Cerner) and mobile app for frontline providers. While our model provides foundational risk stratification, next-generation AI/ML tools could enhance Nocardia management by: (1) Automated diagnostic pipelines: Integrating radiology AI with rapid genomic sequencing; (2) Adaptive prediction systems: Updating risk scores in real-time using ICU telemetry data; (3) Global knowledge synthesis: Federated learning across 50 + hospitals (NOCARDIA-AI initiative).

The nomogram offers a cost-effective and readily implementable tool for clinical practice, requiring only routine data (albumin, imaging findings, and antibiotic records) without additional testing. Its application involves three simple steps: (1) assigning points for each predictor, (2) summing points to stratify patients into low (<30% risk), moderate (30–60%), or high-risk (>60%) categories, and (3) guiding therapy escalation (e.g., dual antibiotics for moderate risk, carbapenems for high risk). Pilot data indicate clinicians can complete scoring in <3 min with high satisfaction (92%). Model adoption may reduce costs by decreasing unnecessary ICU stays (saving ∼$4,200/patient) and broad-spectrum antibiotic use (saving $320/patient). An EHR-integrated version is under development to further streamline workflow. While promising, broader validation across healthcare settings is warranted to assess generalizability, particularly in resource-limited areas with variable imaging access.

The key take-home message from our study is threefold: First, nutritional status (albumin) and specific complications (cavity, empyema) at presentation are more powerful prognostic indicators than traditional immune parameters for *Nocardia farcinica* pneumonia. Second, the initial empiric antibiotic choice significantly impacts outcomes. Most importantly, we synthesize these insights into a validated nomogram that is ready for clinical use. This tool empowers clinicians to move from generalized understanding to personalized prediction, enabling early identification of high-risk patients for whom aggressive nutritional support and tailored antibiotic regimens could potentially improve survival and outcomes.

However, some limitations in this study should be considered. Firstly, although our model shows a high accuracy rate (with an AUC greater than 0.83) and powered for moderate effects, due to the rarity of *Nocardia farcinica* pneumonia cases, the sample size is still limited. The single-center design and lack of external validation may affect generalizability, particularly to: (1) regions with distinct *Nocardia farcinica* strain distributions, (2) immunocompromised populations underrepresented in our cohort (e.g., transplant recipients), and (3) healthcare systems utilizing alternative antibiotic protocols. External validation is needed in a larger sample group to further confirm its clinical applicability. Secondly, while our study examined multiple risk factors, certain potentially important variables were not assessed. Genetic polymorphisms influencing host susceptibility and drug response, as well as environmental exposures (e.g., occupational hazards), may significantly impact disease progression and treatment outcomes. These unmeasured factors could affect model generalizability across different populations and regions.

In conclusion, this study identified key predictors (albumin, empyema, cavitation, and antibiotic regimens) of treatment response in *Nocardia farcinica* pneumonia and developed a clinically useful nomogram. While further multicenter validation is needed, this model provides an actionable tool for risk stratification and treatment optimization, advancing toward personalized management of this complex infection.

## Data Availability

The original contributions presented in this study are included in this article/supplementary material, further inquiries can be directed to the corresponding authors.

## References

[1] Giacobbe D Dettori S Di Pilato V Asperges E Ball L Berti E Pneumocystis jirovecii pneumonia in intensive care units: a multicenter study by ESGCIP and EFISG. *Crit Care*. (2023) 27:323. 10.1186/s13054-023-04608-1 37620828 PMC10464114

[2] Lécuyer R Issa N Camou F Lavergne R Gabriel F Morio F Characteristics and prognosis factors of Pneumocystis jirovecii pneumonia according to underlying disease: a retrospective multicenter study. *Chest*. (2024) 165:1319–29. 10.1016/j.chest.2024.01.015 38215935

[3] Park J Curtis J Jun K Kim T Heo D Ha J Primary prophylaxis for Pneumocystis jirovecii pneumonia in patients receiving rituximab. *Chest*. (2022) 161:1201–10. 10.1016/j.chest.2021.11.007 34788668

[4] Nadig T Thomas N Nietert P Lozier J Tanner N Wang Memoli J Guided bronchoscopy for the evaluation of pulmonary lesions: an updated meta-analysis. *Chest*. (2023) 163:1589–98. 10.1016/j.chest.2022.12.044 36640994 PMC10925546

[5] Prosty C Katergi K Sorin M Rjeily M Butler-Laporte G McDonald E Comparative efficacy and safety of Pneumocystis jirovecii pneumonia prophylaxis regimens for people living with HIV: a systematic review and network meta-analysis of randomized controlled trials. *Clin Microbiol Infect*. (2024) 30:866–76. 10.1016/j.cmi.2024.03.037 38583518

[6] McDonald E Afshar A Assiri B Boyles T Hsu J Khuong N Pneumocystis jirovecii pneumonia in people living with HIV: a review. *Clin Microbiol Rev*. (2024) 37:e0010122. 10.1128/cmr.00101-22 38235979 PMC10938896

[7] Burzio C Balzani E Corcione S Montrucchio G Trompeo A Brazzi L. Pneumocystis jirovecii Pneumonia after heart transplantation: two case reports and a review of the literature. *Pathogens*. (2023) 12:1265. 10.3390/pathogens12101265 37887781 PMC10610317

[8] Sierra C Daiya K. Prophylaxis for Pneumocystis jirovecii pneumonia in patients with inflammatory bowel disease: a systematic review. *Pharmacotherapy*. (2022) 42:858–67. 10.1002/phar.2733 36222368 PMC9828113

[9] Parker N Cheng W Hindley G O’Connell K Karthikeyan S Holen B Genetic overlap between global cortical brain structure, C-reactive protein, and white blood cell counts. *Biol Psychiatry*. (2024) 95:62–71. 10.1016/j.biopsych.2023.06.008 37348803 PMC11684752

[10] Arenas-Jiménez J García-Garrigós E Ureña Vacas A Sirera Matilla M Feliu Rey E. Organizing pneumonia. *Radiologia*. (2022) 64:240–9. 10.1016/j.rxeng.2022.08.002 36737163

[11] Trabelsi B Ghorbel S Ben Rabeh R Bouassida M Ben Ali M. C- reactive protein in the early diagnosis of pneumonia complicating severe blunt chest trauma. *Tunis Med*. (2023) 101:756–8. 38465756 PMC11261490

[12] Kunutsor S Laukkanen J. Serum C-reactive protein-to-albumin ratio is a potential risk indicator for pneumonia: findings from a prospective cohort study. *Respir Med*. (2022) 199:106894. 10.1016/j.rmed.2022.106894 35659744

[13] Cappelli S Casto E Lomi M Pagano A Gabbrielli L Pancani R Pleural effusion in COVID-19 pneumonia: clinical and prognostic implications-An observational, retrospective study. *J Clin Med*. (2023) 12:1049. 10.3390/jcm12031049 36769697 PMC9917650

[14] Tang J Ouyang Q Li Y Zhang P Jin W Qu S Nanomaterials for delivering antibiotics in the therapy of pneumonia. *Int J Mol Sci*. (2022) 23:15738. 10.3390/ijms232415738 36555379 PMC9779065

[15] Moskalik M. Sulfonamides with heterocyclic periphery as antiviral agents. *Molecules*. (2022) 28:51. 10.3390/molecules28010051 36615245 PMC9822084

[16] Silva A Melro L Besen B Mendes P Park M. Sulfonamide-induced acute eosinophilic pneumonia requiring extracorporeal membrane oxygenation support: a case report. *Crit Care Sci*. (2023) 35:239–42. 10.5935/2965-2774.20230404-en 37712817 PMC10406408

[17] Mo Y Booraphun S Li A Domthong P Kayastha G Lau Y Individualised, short-course antibiotic treatment versus usual long-course treatment for ventilator-associated pneumonia (REGARD-VAP): a multicentre, individually randomised, open-label, non-inferiority trial. *Lancet Respir Med*. (2024) 12:399–408. 10.1016/S2213-2600(23)00418-6 38272050

[18] Dimitropoulos D Karmpadakis M Paraskevas T Michailides C Lagadinou M Platanaki C Inflammatory biomarker-based clinical practice in patients with pneumonia: a systematic review of randomized controlled trials. *Rom J Intern Med*. (2024) 62:241–59. 10.2478/rjim-2024-0013 38536775

